# Hydroquinone: Environmental Pollution, Toxicity, and Microbial Answers

**DOI:** 10.1155/2013/542168

**Published:** 2013-07-15

**Authors:** Francisco J. Enguita, Ana Lúcia Leitão

**Affiliations:** ^1^Instituto de Medicina Molecular, Faculdade de Medicina, Universidade de Lisboa, Avenida Prof. Egas Moniz, 1649-028 Lisboa, Portugal; ^2^Departamento de Ciências e Tecnologia da Biomassa, Faculdade de Ciências e Tecnologia, Universidade Nova de Lisboa, Quinta da Torre, Campus de Caparica, 2829-516 Caparica, Portugal

## Abstract

Hydroquinone is a major benzene metabolite, which is a well-known haematotoxic and carcinogenic agent associated with malignancy in occupational environments. Human exposure to hydroquinone can occur by dietary, occupational, and environmental sources. In the environment, hydroquinone showed increased toxicity for aquatic organisms, being less harmful for bacteria and fungi. Recent pieces of evidence showed that hydroquinone is able to enhance carcinogenic risk by generating DNA damage and also to compromise the general immune responses which may contribute to the impaired triggering of the host immune reaction. Hydroquinone bioremediation from natural and contaminated sources can be achieved by the use of a diverse group of microorganisms, ranging from bacteria to fungi, which harbor very complex enzymatic systems able to metabolize hydroquinone either under aerobic or anaerobic conditions. Due to the recent research development on hydroquinone, this review underscores not only the mechanisms of hydroquinone biotransformation and the role of microorganisms and their enzymes in this process, but also its toxicity.

## 1. Introduction

Industrial development has caused a huge increase in the release of potentially toxic compounds into the atmosphere, water bodies, and soils. In the last decades, environmental pollutants have been directly connected to the increase in human diseases, particularly those involved with the immune system. The contribution of benzene and its metabolites to this issue is well recognized, making them a public health problem. 

Hydroquinone, the major benzene metabolite, is a ubiquitous chemical in the environment due to its widespread application in human and industrial activities. It can be used as a developing agent in photography, dye intermediate, stabilizer in paints, varnishes oils, and motor fuels. In addition, hydroquinone has been used as an antioxidant in the rubber and food industry. From 1950s to 2001 hydroquinone was applied in the commercially available cosmetic skin lightening formulations in European Union countries and since 1960s it was commercially available as a medical product. It is also present in cosmetic formulations of products for coating finger nails and hair dyes [1]. On the other hand, hydroquinone can be a component of high molecular aromatic compounds (e.g., resin), an intermediate, or appear as a degradation product generated by transformation of aromatic compounds. Advanced oxidation processes (APOs) of aromatic compounds, particularly of phenol, yield several benzene derivatives, such as hydroquinone, catechol, and resorcinol, as intermediate metabolites of its transformation. The formation of hydroquinone and *ρ*-benzoquinone at early stages of phenol oxidation increases the toxicity of phenol wastewaters, showing that these compounds were more toxic and less degradable than the original pollutant [2]. Meanwhile, in the oxidative degradation of hydroquinone under a supercritical condition (409.9°C and 24.5 MPa) and subcritical condition (359.9°C and 24.5 MPa), *ρ*-benzoquinone was to be an important intermediate [3]. Despite the toxic properties, a number of microorganisms can utilize hydroquinone, especially under aerobic conditions, which has led to the development of low-cost treatment of polluted effluents. The chemical method applied conventionally to the treatment of industrial wastewater used FeSO_4_ and H_2_O_2_; however, the application of this technology generates ferric sulfate, which enables recycled reactants [4]. Therefore, biological transformations are generally preferred for being considered as more economical and environmentally friendly.

Fungi as well as bacteria are known to be capable of transforming or mineralizing hydroquinone; *Aspergillus fumigatus, Candida parapsilosis, Tyromyces palustris, Gloeophyllum trabeum, Penicillium chrysogenum,* and *Phanerochaete chrysosporium* are examples of fungi able to degrade hydroquinone [5–9]. While several bacteria such as *Pseudomonas* sp., *Alcaligenes* sp., and *Moraxella* sp. are capable of utilizing this aromatic compound [10–15], there are a considerable number of studies about the toxic effects of hydroquinone. However, many of these studies have been conducted very recently, showing the *in vivo* hydroquinone toxicity exposure effects [16–20]. Hence, the aims of the present review are firstly to outline the toxic effects of hydroquinone and secondly to present an overview of the role of microorganism in the degradation of hydroquinone.

## 2. Properties 

Hydroquinone is an aromatic compound consisting of the benzene ring and two –OH groups at *para* position. It is available in the form of white crystals, but industrial use grades may be light grey or light tan. Contact with air and light causes oxidation and darkening of color. Hydroquinone is soluble in water, methanol, and ether. However, it has less solubility in water than the other two dihydroxybenzenes, which means hydroquinone has less affinity towards hydrophilic solvents. Its octanol/water partition value is also less than that of catechol and resorcinol (Table 1). 

Hydroquinone can occur naturally in many plant foods, as glucose conjugate, namely, arbutin, for example, in the wheat, pears, coffee, onion, tea and red wine [21]. Arbutin is readily hydrolyzed in the stomach to free hydroquinone, which is widely absorbed by the gastrointestinal tract [22].

Hydroquinone is autoxidized by two successive one-electron oxidations, producing an extremely reactive semiquinone intermediate, which is the most reactive and most toxic intermediate of the quinone species. Dihydroxybenzene and quinones are recognized to induce oxidative stress as well as to nonspecifically bind both DNA and protein [23]. Hydroquinone can form complexes with various di- and trivalente metal ions, such as copper and iron. In the case of copper, the complex formed increased H_2_O_2_ production by hydroquinone and enhances its autooxidation to benzoquinone [24].

Hydroquinone can be originated during phenol [30] or benzene biotransformation [31]. The benzene is first metabolized by liver cytochrome P-450 monooxygenase to phenol. Further hydroxylation of phenol by cytochrome P-450 monooxygenase or by human peroxidase resulted in the formation of mainly hydroquinone, which accumulates in the bone marrow [32]. 

 Hydroquinone can also be produced through three chemical processes, involving oxidation, reduction, and alkylation reactions. Firstly, it can be generated by oxidation of phenol; secondly, the oxidation of aniline with manganese dioxide in acidic conditions, followed by reduction with iron dust in aqueous medium; finally, the alkylation of benzene with propylene to originate the *para*-di-isopropylbenzene isomer, besides other isomers, which is oxidized and produces the corresponding dihydroperoxide, that is subsequently treated with an acid to originate hydroquinone [28].

## 3. Toxicity

### 3.1. Toxicity to Aquatic and Soil Organisms

It is known that phenolic compounds are extremely toxic for aquatic organisms at the concentration level of part-per-million and most of them can influence the organolectic properties of shellfish and fish at part-per-billion level [33]. In fact, hydroquinone is highly toxic to aquatic organisms, such as *Pimephales promelas, Brachydanio rerio, Daphnia magna, Desmodesmus armatus, Synechocystis *sp., *Nostoc *sp., and *Microcystis aeruginosa* [26, 34]. The acute 48 h EC_50_ value of 0.15 mg/L for the marine *Daphnia magna*, and 24 h LC_50_ values ranging from 0.22 to 0.28 mg/L for *Brachionus plicatilis* have been reported [33]. Studies on *Photobacterium phosphoreum* showed that hydroquinone is one hundred and one thousand times more toxic than catechol and resorcinol, respectively [35]. Meanwhile, it was reported that hydroquinone was the less toxic dihydroxybenzene to the gram-positive bacteria *Bacillus subtilis*; however, it was shown that hydroquinone and catechol mixture exerts a synergistic joint action while the other mixtures have an additive actions [36].

The toxic effect of phenolic compounds on soil microbial activity has been evaluated, showing hydroquinone as the most toxic dihydroxybenzene [37]. The number of cultivable microorganisms decreased with increasing concentration of phenolic compounds. Furthermore, it was suggested that the low dehydrogenase and *β*-glucosidase activity found in the soils treated by hydroquinone and catechol was due to their low water soluble carbon concentration and high inhibitory effects, respectively [37].

Hydroquinone generally gives a negative response in the standard bacterial gene mutations studies, such as Ames' test [38]. Ames' test showed that hydroquinone was not mutagenic for *Salmonella typhimurium *strain TA 98, TA 100, TA 1535, and TA 1537 [39], while in yeast cells it was reported that the exposure to 1,4-dihydroxybenzene increases homologous recombination [40]. It has also been suggested that hydroquinone induces aneuploidy in *Saccharomyces cerevisiae.* Shiga et al. [38] reported in *Saccharomyces cerevisiae* that the hydroquinone induced G2/M transition checkpoint, which is activated by the Hog1-Swe1 pathway, having a role in the formation of aneuploidy. Functional studies* in vitro *in yeast cells lacking the DNA helicase Sgs 1p, required for the maintenance of genomic stability, shown reduced cellular growth in the presence of hydroquinone, and RNAi knockdown of *WRN*, the human ortholog of *SGS1*, increases hydroquinone generated DNA damage, particularly at high doses of dihydroxybenzene [41]. Later, North et al. [23] postulated that Pst2p and Ycp4p, putative mitochondrial NAD(P)H:quinone oxidoreductases, to be novel yeast orthologs of NQO1 (NAD(P)H:quinone oxidoreductase 1 of humans) that are required for hydroquinone tolerance. 

### 3.2. Toxicity to Mammalian and Human Cells

It has been postulated that the toxicity of hydroquinone could have been underestimated taking into account the positive data from a limited number of confirmations in experimental animals and the inconclusive evidence in humans [27]. Indeed, it has been reported that hydroquinone induces mononuclear cell leukemia, renal tubular cell tumors, and liver cancer in rodents [42]. Tsutsui and colleagues provided pieces of evidence that hydroquinone has cell transforming and genotoxic activity over mammalian cells in culture. After cell treatment with hydroquinone, the frequencies of DNA gaps, breaks, and sister chromatid exchanges were increased as well as chromosome aberrations [31]. Moreover, a combination of the hydroquinone, catechol, and phenol is shown to act synergistically, triggering oxidative DNA damage and genotoxicity in mammalian cells *in vivo* [43].

Hydroquinone has been shown to be a potential toxic agent that influences immune cell responses. It increases allergic immune responses through the increase in interleukin(IL)-4 production and immunoglobulin E (IgE) levels [44]. Recently, it has been reported that low levels of *in vivo* hydroquinone in mice, which is not responsible for myelotoxicity, activate oxidative stress and membrane receptors in circulating neutrophils, contributing to the impaired innate host protection against bacteria [16]. Later, it has been shown that hydroquinone affects the lipopolysaccharide induced cytokine secretion and nitric oxide production by neutrophils by interfering with pretranscriptional and posttranscriptional mechanisms [18]. Shimada et al. [20] proposed the mechanism of *in vivo *hydroquinone toxicity by exposing mice at low levels of dihydroxybenzene by inhalation. Their findings showed that *in vivo* hydroquinone exposure impairs circulating mononuclear cell migration into the inflamed area. It was also suggested that the direct inhibitory action of hydroquinone on monocyte chemoattractant protein-1 (MCP-1) production by lung cells is directly related to the impaired mononuclear cell chemotaxis [20].

In cultured human cells, induction of DNA strand breaks was dependent on the presence of copper(II) ions [45]. It was proposed that hydroquinone leads to DNA damage through peroxide production in cells, prior internucleosomal DNA fragmentation leading to apoptosis [45]. Luo and coworkers reported in human hepatoma HepG2 cells that hydroquinone caused DNA strand breaks as well as DNA-protein crosslinks and chromosome breaks. Further, they postulated that hydroquinone exerts genotoxic effects in HepG2 cells through DNA damage by oxidative stress, being glutathione the responsible for cellular defense against dihydroxybenzene effects [46]. There are several consistent results on the effect of hydroquinone in the induction of sister chromatid exchange in human lymphocytes *in vitro *[47–49]. Meanwhile, hydroquinone seems to modulate immune responses, since it inhibits lymphocyte proliferation by suppression of DNA synthesis [50] and exerts a cytotoxic effect in neutrophils, eosinophils, and lymphocytes via caspase 9/3 pathway [17, 19].

## 4. Biodegradation and Biotransformation 

Fungi and bacteria can both degrade and transform phenolic compounds. Filamentous fungi have the advantage to be able to translocate resources, such as nutrients and water, between different parts of their mycelium, which could be essential for the transformation or detoxification of phenolic compound. Microorganisms are, therefore, looked upon as an effective method of removing these pollutants. Hydroquinone can be either the reagent or the product of a transformation process. First we will describe processes where hydroquinone is produced from other phenolic compounds. Next, we will review the enzymes and pathways involved in hydroquinone catabolism. 

Degradation of hydroquinone by fungi has been reported. The benzaldehyde and benzoic acid metabolism by the brown-rot basidiomycetes *Tyromyces palustris* and *Gloeophyllum trabeum *led to the formation of hydroquinone, which it is further metabolized. Kamada and coworkers reported that hydroquinone was metabolized, but no formation of products was observed. Indeed, the same authors described the effective mineralization of aromatic compound by the brown-rot fungi using radioactive substrates [7]. The hydroxylated intermediate was also found as product of phenol metabolism of fungi. The ascomycetous fungi, *Penicillium chrysogenum *var. *halophenolicum *(previously known as *Penicillium chrysogenum *CLONA2) is able to complete mineralization of phenol in single and combined phenol and glucose cultures. However, during the conversion of phenol in the combined phenol and glucose cultures, hydroquinone was accumulated in the early stages of incubation and disappeared after 80 hours of culture, indicating that hydroquinone was a metabolic intermediate, but it is not a dead-end product [8]. It has been also detected in the biodegradation of 4-ethylphenol by *Aspergillus fumigates,* another ascomycetous fungi. According to these authors, hydroquinone was obtained by hydrolysis of 4-hydroxyphenylacetate, which undergoes further hydroxylation to form 1,2,4-trihydroxybenzene followed by ring fission substrate to produce maleylacetate [5]. 

Several aerobic bacteria are capable of utilizing hydroquinone as a product compound obtained from other substrate, involving a hydroquinone 1,2-dioxygenase to convert hydroquinone into 4-hydroxymuconic semialdehyde. Degradation of 4-chlorophenol via hydroquinone pathway has been reported for several strains belonging to the actinobacterium group. *Arthrobacter ureafaciens *CPR706 first eliminates the chloro-substituent to form hydroquinone. The CPR706 strain also degrades other *para*-substituted phenols, including 4-fluoro, 4-bromo, 4-iodo, and 4-nitrophenol via the hydroquinone pathway [51]. According to Zhang and coworkers, the hydroquinone pathway is also used in *para-*nitrophenol degradation by gram-negative bacteria such as *Moraxella *sp. [10], *Pseudomonas *sp. strain WBC-3 [13], and *Pseudomonas *sp. 1–7 [15]. It has been reported that *Pseudomonas fluorescens *ACB is able to use 4-hydroxyacetophenone through the initial formation of 4-hydroxyphenyl acetate and hydroquinone [11, 12, 52, 53]. Meanwhile, a second pathway branch was established for 4-chlorophenol transformation into hydroquinone, which in turn was hydroxylated to originate hydroxyquinol [54].

## 5. Key Enzymatic Players in Hydroquinone Biodegradation and Metabolism

**Figure 1:**
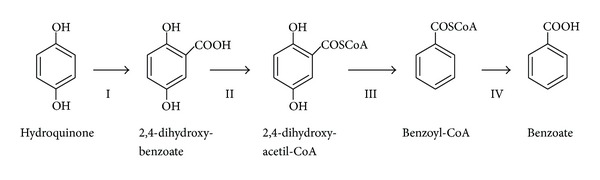
Anaerobic pathway for the metabolization of hydroquinone. I: hydroquinone carboxylase; II: hydroquinone Acetil-CoA transferase; III: benzoyl-CoA oxidoreductase; IV: benzoyl-CoA hydrolase.

Hydroquinone can be degraded by two different pathways depending on the oxygen availability. However, the anaerobic metabolization of hydroquinone is a less frequent process in nature, mainly restricted to a specific group of bacteria. It involves the conversion of hydroquinone to benzoate with an intermediate carboxylation, and activation of the products by their linkage to acetyl-CoA (Figure 1). Cells can either employ benzoate as an anabolic fundamental brick or introduce the CoA-activated metabolites in the beta-oxidative catabolic pathway.

**Figure 2:**
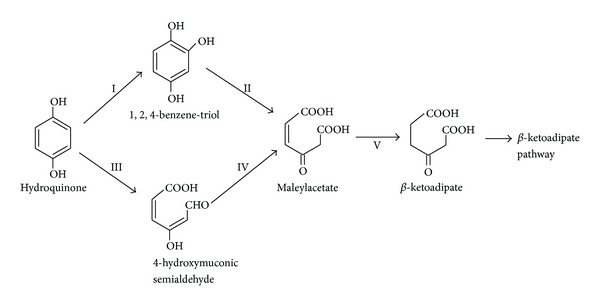
Two different branched pathways for the biodegradation of hydroquinone under aerobic conditions. I: hydroquinone hydroxylase; II: 1,2,4-trihydroxybenzene 1,2-dioxygenase; III: hydroquinone dioxygenase; IV: 4-hydroxymuconic semialdehyde dehydrogenase; V: beta-ketoadipate oxidoreductase.

In aerobic conditions hydroquinone is channeled to the beta-ketoadipate pathway through two different metabolic branches (Figure 2). The first pathway involves the initial hydroxylation of hydroquinone to 1,2,4-trihydroxybenzene followed by a ring-fission reaction catalyzed by a 1,2-dioxygenase [55–57]. The second pathway of hydroquinone degradation is less common in nature. In this pathway, hydroquinone ring is directly cleaved by a specific hydroquinone 1,2-dioxygenase and the generated semialdehyde oxidized to maleylacetate [10, 58]. The first aerobic branch has been characterized in bacteria and fungi; meanwhile, the second is exclusive of prokaryotic organisms.

## 6. Degradation of Hydroquinone under Aerobic Conditions

### 6.1. Catalysis of Direct Aromatic Ring Fission: 1,2-Hydroquinone Dioxygenase

Hydroquinone 1,2-dioxygenases (HQDIOX) are mainly bacterial enzymes able to catalyze the direct phenolic ring fission using 1,4-dihydroxyphenols as substrates and constituting a wide new family of aromatic ring-fission enzymes. Their catalytic capabilities made them an excellent tool for phenol bioremediation. HQDIOX can be classified within two subfamilies of enzymes: monomeric nonheme iron containing enzymes [59], and two-component dioxygenases, that were originally characterized as members of the gene clusters involved in the degradation of phenolic pollutants in *Pseudomonas* [11, 12].

Monomeric hydroquinone 1,2-dioxygenases were first characterized as a core catalytic members of the enzymatic system responsible for the degradation of some pollutants as pentachlorophenol (PCP).

**Figure 3:**
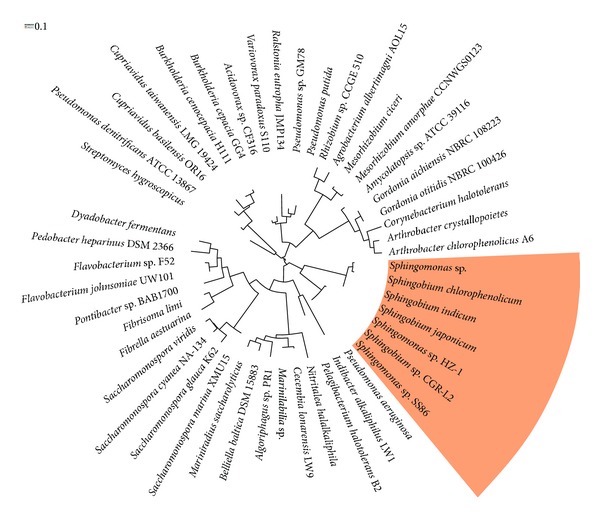
Phylogenetic circular cladogram of hydroquinone 1,2-dioxygenase sequences in diverse bacteria. Species from the genera *Sphingomonas *and *Sphingobium* are shadowed.

These single chain enzymes were first described in *Mycobacterium chlorophenolicum*, *Sphingobium chlorophenolicum,* and *Novosphingobium *[59–62]. In *S. chlorophenolicum* the HQDIOX is encoded by the *pcp*A gene, which encodes a 37 kDa protein. PcpA protein contains a nonheme Fe atom and has no homology with classical ring-fission enzymes such as catechol dioxygenase. The enzyme is able to catalyze the ring-opening of a wide range of substituted hydroquinones [60, 63, 64]. In spite of the detailed knowledge of the PcpA enzyme from *S. chlorophenolicum*, other putative members of the family are present in several gram-negative bacteria (Figure 3).

**Figure 4:**
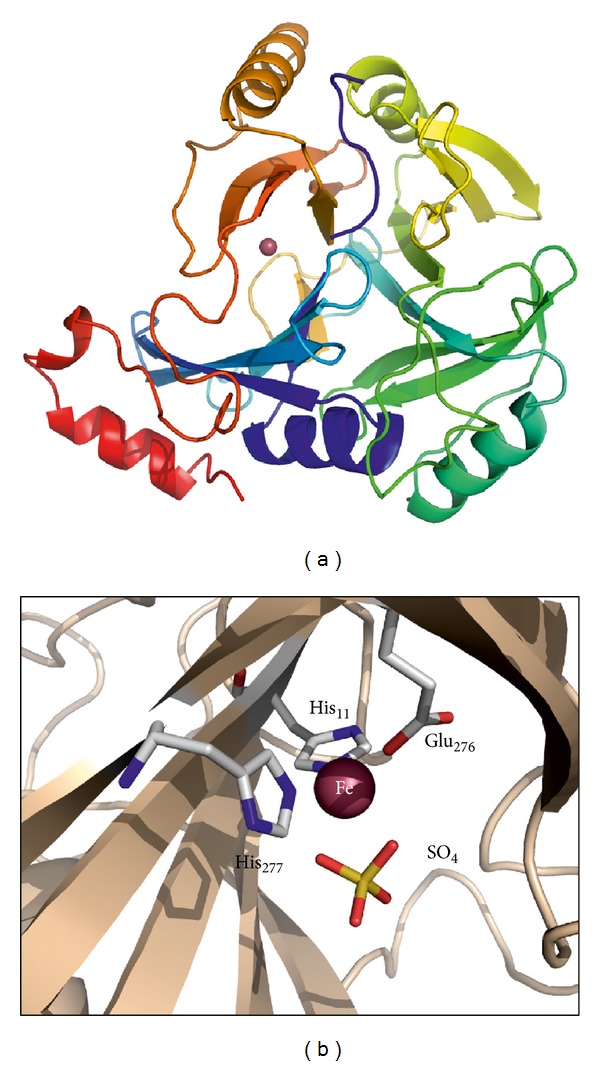
Structural features of the hydroquinone 1,2-hydroxylase from *S. chlorophenolicum*, as determined by X-ray crystallography (PDB code: 4HUZ). (a) Overall folding of the enzyme depicting the central position of the iron atom; (b) close view of the coordinating residues and the Fe atom environment. The sulfate ion close to the iron atom comes from the crystallization media.

The catalytic heart of the PcpA enzyme is composed of a non-heme iron atom coordinated by two histidines and an aspartate (Figure 4) as observed in the crystal structure of the PcpA enzyme from *S. chlorophenolicum* that has been recently determined [64]. The iron atom is located in a surface cleft of the protein limited by two groups of antiparallel beta-strands. The observed structure of PcpA suggested a potential catalytic mechanism, which will be distinct from the classical extradiol dioxygenases. In these enzymes, the 1,4-hydroxyl groups of the phenolic substrate seem essential for affinity, thus other phenols such as catechol, nitro-catechol, phenol, bromo, and chlorophenols did not show any apparent affinity for the PcpA protein [64].

Another family of hydroquinone 1,2-dioxygenases, completely unrelated to PcpA, has been characterized by *Sphingomonas* and *Pseudomonas *with putative homologs also present in the genomes of *Photorhabdus*, *Burkholderia*, and *Variovorax *genera. They are composed of two subunits encoded by adjacent genes in genomic clusters involved in the degradation of substituted aromatic compounds. In *Sphingomonas* sp. these genes are designated as *hdq*A and *hdq*B [65], and the corresponding homologs in *P. fluorescens* are *hap*C and *hap*D [11, 12]. In *P. fluorescens*, the encoding genes are located in a cluster composed of *hapCDEFGHIAB*, responsible for the biodegradation of hydroxylacetophenone [11, 12]. The purified enzyme from *P. fluorescens* is a heterotetramer with a quaternary structure of *α*_2_ *β*_2_, with a molecular weight of 115 kDa. The beta-subunit, encoded by *hap*D gene, has a molecular weight of 38 kDa, contains a coordinated nonheme iron, and is also responsible for the substrate binding. The enzyme from *P. fluorescens* can act over a wide range of hydroquinone derivatives including 2-chloro-hydroquinone, 2-methyl-hydroquinone, 2-methoxy-hydroquinone, and 2-ethyl-hydroquinone. The enzymatic activity over aliphatic derivatives with longer chains located in the *ortho* position of the benzene ring is almost undetectable, indicating the presence of a tight substrate pocket and steric clash interactions with the substrates of higher molecular weights. Moreover, the enzyme is not inhibited by monophenols, but the activity is strongly decreased with the presence of cyanide compounds, as expected for an iron-dependent enzyme [11, 12, 53]. Apparently, the iron atom is in the core of the catalytic mechanism that could be essentially similar to the one postulated in the monomeric enzymes; however, the role of the alpha small subunit of the enzyme remains elusive and will require further investigation. 

### 6.2. 4-Hydroxymuconic Semialdehyde Dehydrogenase

This enzyme, responsible for the conversion of 4-hydroxymuconic acid into maleylacetate, has been described in *Pseudomonas* and *Sphingomonas *genera and is encoded by genes associated to the degradation of *p*-nitrophenol and hydroquinone [15, 65]. In *Pseudomonas*, the enzyme is encoded by the *hapE* gene, which encodes a protein of 487 amino acids and a calculated molecular weight of 50 kDa. Other homologs of HapE protein are also present in *Burkholderia* sp., *Sphingomonas* sp., *Azospirillum amazonense*, and in *Brachymonas petroleovorans*. The enzyme is an oxidoreductase that used NADP nucleotides as electron acceptors [11, 12, 66]. It belongs to the NAD(P)-dependent aldehyde dehydrogenase superfamily, a group of enzymes with an important role either in detoxification reactions or in other metabolic pathways.

### 6.3. Hydroquinone Hydroxylases: A Member of a Complex Family of Enzymes

Hydroquinone hydroxylase belongs to the family of phenol 2-monooxygenases showing wider substrate specificity when compared with hydroquinone dioxygenases. This enzymatic activity has been extensively characterized in anamorphic yeasts as *Trichosporon cutaneum* [67, 68] and *Candida *[6, 69] as well as in several species of gram-negative bacteria [65, 70]. 

Phenol oxygenases are classified into two main groups: one family of monomeric enzymes and another one composed of multicomponent proteins. Interestingly, genes encoding monomeric hydroquinone hydroxylases are typically encoded in plasmid DNA, whereas the multicomponent proteins are encoded by genes located in the bacterial chromosome. The monomeric enzymes are environmentally relevant due to their presence in mobile genetic systems that can be transferred between different cells. In bacteria, hydroquinone hydroxylase activity has been associated to monomeric iron-containing metalloenzymes. In *Acinetobacter* and *Pseudomonas* the enzyme containing an iron-sulfur cluster of the type 2Fe-2S [71] is responsible for the electron transfer from the substrate to the reduced cofactor NADH [72, 73]. 

**Figure 5:**
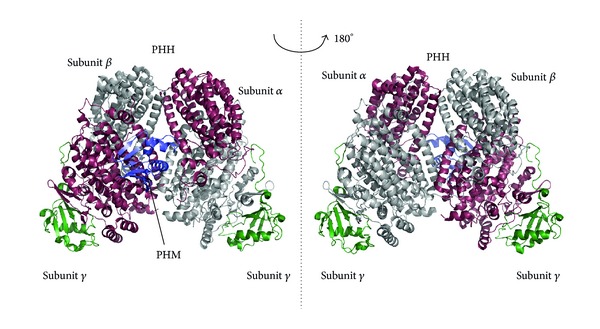
Ribbons representation of the overall tridimensional structure of the catalytic core PHH of the phenol monooxygenase from *Pseudomonas* sp. in complex with the regulatory subunit PHM (PDB code: 2INN). The catalytic core of the enzyme is composed of a dimer of three subunits *αβγ* [73, 74].

Moreover in *Pseudomonas*, another family of phenol monooxygenases able to use hydroquinone as a substrate and belonging to the multicomponent group has been also described. Bacterial multicomponent monooxygenases (BMMs) consist of a protein complex with 250–300 kDa complex containing a dimeric hydroxylase (PHH) of the form (*αβγ*)_2_, a small regulatory protein (PHM) and a flavoprotein containing an iron-sulphur cluster (PHP), which acts as reductase supplying electrons to the hydroxylase component and consuming NADH as a cofactor. In spite of their structural complexity (Figure 5), multicomponent phenol monooxygenases are extremely efficient enzymes, able to act over a wide range of substrates as nitro, amino, and methyl-phenols and also halogenated phenols [74, 75]. They are typically inhibited by their substrates and also by inorganic anions such as nitrite, sulfate, and phosphate, showing a slightly alkaline optimum pH. The complexity of the multicomponent phenol monooxygenase is still far from being understood, since many additional protein components have been recently discovered. In fact genes encoding BMMs are located in a genomic cluster. Accessory components such as PHK protein have been described as enhancers of the overall catalytic activity of the complex [70].

In eukaryotes, phenol hydroxylases with activity over hydroquinone are enzymes encoded by chromosomic genes, with a molecular weight of about 150 kDa, and acting as functional dimers. Hydroquinone hydroxylase from *Candida parapsilosis* and phenol hydroxylase from *Trichosporon cutaneum* are ClassA flavoprotein monooxygenases [76]. In yeast hydroquinone hydroxylase activity is induced when the cells are grown on mono- or dihydroxy benzoic acids as a sole source of carbon and energy [6].

**Figure 6:**
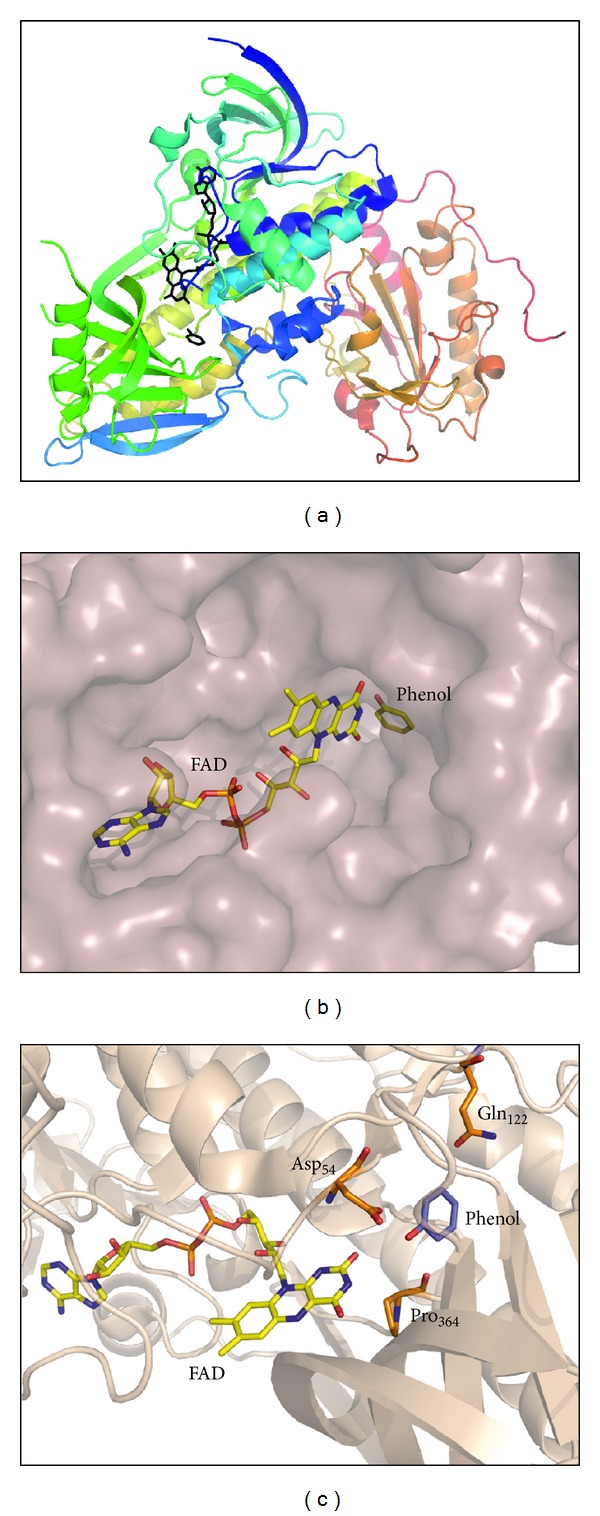
Structure of phenol hydroxylase from *T. cutaneum* as determined by X-ray crystallography (PDB code: 1PN0), an enzyme also able to act over hydroquinone. (a) Overall fold showing the structure of the three structural domains and the location of FAD cofactor and phenolic substrate in the catalytic pocket of the enzyme between domains 1 and 2; (b) detailed view of the catalytic pocket, showing the phenolic substrate close to the FAD cofactor; (c) close-up view of the residues involved in the formation of a narrow substrate hole that accommodates monophenols, and located in a flexible protein region that also interacts with the FAD cofactor. The substrate-cofactor pocket is composed of a continuous cavity in the protein body, which is closed by the flavin ring of the FAD cofactor avoiding the escape of the substrate.

Hydroquinone hydroxylase contains a tightly noncovalently bound FAD cofactor per each monomer, which is essential for the physiological reconstitution of the apoenzyme (Figure 6) [6]. Structural studies, performed by X-ray crystallography in the *T. cutaneum* hydroquinone hydroxylase, showed that the protein is composed of three clearly defined domains (Figure 6). The first domain is composed of a beta-sheet which forms the FAD binding site and also the substrate pocket close to the interface with the second domain. In the third domain a thioredoxin-like fold is responsible for the protein-protein interactions that built up the apoenzyme dimer [77]. Eukaryotic phenol hydroxylases are able to act over simple phenols, over amino, halogen as well as methyl-phenol derivatives with the only requirement of having a free *ortho* position in the phenolic ring. 

### 6.4. 1,2,4-Trihydroxybenzene 1,2-Dioxygenase

Also known as hydroxyquinol 1,2-dioxygenase (1,2-HQD), 1,2-HDQs belong to the intradiol dioxygenase family and catalyze the oxidative ring cleavage of substituted 1,2-dihydroxy-benzenes. In bacteria and fungi, these enzymes are homodimers composed of two identical subunits of 30–35 kDa, tightly packed that suggests a possible allosteric interaction between catalytic monomers. They have been described in gram-negative bacteria (*Burkholderia cepacia*, *Azotobacter* sp., and *Ralstonia pickettii*), gram-positive bacteria (*Nocardioides simplex* and *Arthrobacter* sp.), and fungi (*T. cutaneum* and *Phanerochaete chrysosporium*) [51, 78–85]. 

**Figure 7:**
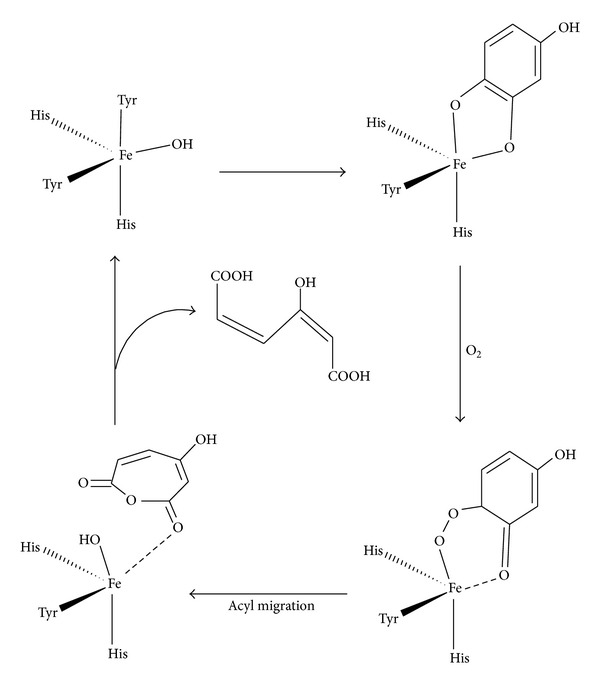
Proposed reaction mechanism for hydroxyquinol intradiol 1,2-dioxygenase. The enzyme catalyzed the ring-fission reaction by generation of a seven-membered intermediate ring using molecular oxygen. (Adapted from [84]).

1,2-HQDs are iron-dependent dioxygenases, containing a nonheme pentacoordinated Fe(III) atom located close to the substrate binding pocket. This catalytic cavity for substrate binding is composed of a tridimensional arrangement of beta-sheets together with a number of random coils that open a small hydrophobic concavity that will position the substrate close to the iron atom. The proposed catalytic mechanism for intradiol 1,2 cleavage has been recently proposed based on the crystallographic structure of 1,2-HQD from *N. simplex* [84]. The enzyme is able to catalyze the aromatic ring fission by a mechanism that involves the formation of an intermediate oxo-adduct and a seven-membered ring (Figure 7).

**Figure 8:**
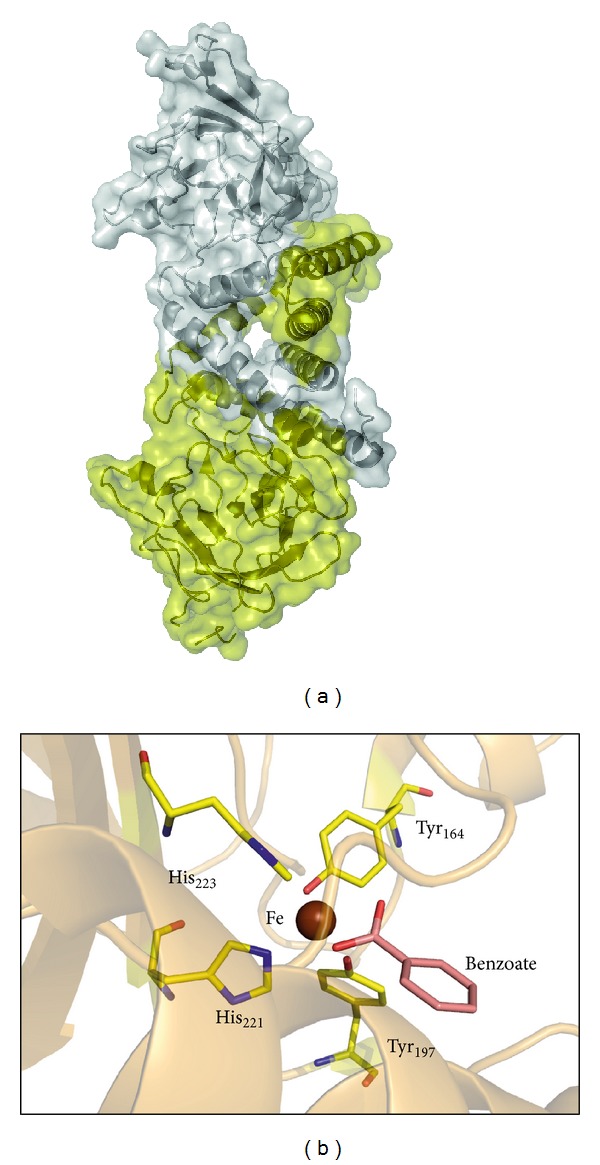
Tridimensional structure of the catalytic homodimer of hydroxyquinol 1,2-dioxygenase from *N. simplex* as determined by X-ray crystallography (PDB code: 1TMX). (a) Global structure of the homodimer showing the tight packing between subunits; (b) close look to the iron center, coordinating amino acids, and substrate binding site close to the iron atom. The structure of hydroxyquinol 1,2-dioxygenase was solved in the presence of benzoate, a competitive inhibitor which binds to the substrate binding site.

As reported in the tridimensional structure of the 1,2-HQD, the substrate specificity of the enzyme is expected to be controlled by the aromatic ring substituents (Figure 8). In fact 1,2-HQD is more similar to the Type I of intradiol dioxygenases, which showed an increased specificity in their substrates [84, 86].

Studies performed with diverse 1,2-HQD enzymes showed a decreased affinity and catalytic activity over catechol, pyrogallol, and other substituted dihydroxybenzenes in comparison with 1,2,4-trihydroxybenzene. Interestingly, the sequence comparison of 1,2-HQDs among diverse microorganisms showed a divergent evolution pattern between these families of enzymes and other catechol dioxygenases, indicating that the development of substrate specificity for hydroquinone was a very important step in the evolution of pathways for the efficient degradation of natural and xenobiotic compounds with a benzene nucleus [83, 87, 88]. 

## 7. Hydroquinone Degradation under Anaerobic Environment

The presence of two hydroxyl groups in *para* orientation within the benzene ring makes improbable that any microorganism would be able to catalyze direct oxidative ring fission of hydroquinone under anaerobic conditions. On the contrary, anaerobic hydroquinone-degrading microorganisms will engage a diverted pathway which involves a carboxylation and activation with CoA to finish with the production of benzoate and the introduction of this compound into the classical anaerobic benzene metabolization pathways [89, 90]. In spite of their bioremediation potential, there are just a few reported examples in the literature describing anaerobic organisms able to degrade hydroquinone. They include sulfate-reducing bacteria from the genus *Desulfococcus* [91, 92] and dehalogenating bacteria isolated from soil consortia together with filamentous fungi [93, 94].

The hydroquinone anaerobic biodegradation pathway starts with a carboxylation to produce gentisate (2,5-dihydroxybenzoate), catalyzed by an uncharacterized carboxylase enzyme that is inducible by the presence of hydroquinone as a sole source of carbon and energy in anaerobic conditions [91, 92]. After the synthesis of gentisate, this compound will be activated by the addition of a CoA group. This is a classical reaction step in the utilization of phenolic compounds by anaerobic microorganisms [95]. The corresponding CoA-ligase involved uses only Acyl-CoA as a donor, with no evidence of catalytic activity using acetyl or phenyl-CoA donors [91, 92]. The most interesting reaction of the whole pathway is the reductive dehydroxylation of the gentisyl-CoA. This reaction is catalyzed by an oxygen-sensitive enzyme, which removes both hydroxyl groups in one single step, and probably associated to cell membranes [90–92]. However, further investigations are required to characterize this enzymatic activity. The gentisyl-CoA dehydroxylase will generate benzoate as a final product that will engage the anaerobic benzoate pathway for its final degradation towards beta-oxidation.

## 8. Final Remarks

Hydroquinone is a highly redox-active compound, which can promote the formation of reactive oxygen species, oxidative stress, and DNA damage. Despite the fact that the molecular modes of action of hydroquinone in disease remain unclear, it has been proposed that it could act in a synergistic way by inducing general DNA damage together with a specific action over the mitotic spindle, and inhibition of topoisomerase II that could result in DNA strand breaks via production of reactive oxygen species (ROS). Recent studies also showed that hydroquinone promotes tumor cell growth and suppresses the immune response.

Many industrial pollutants, as well as natural metabolites, are phenolic compounds, and the degradation of these aromatic molecules is important in what concerns to carbon cycle. The incredible versatility inherited in microorganism either due to its tolerance for extreme conditions or by the capability to adapt its enzymatic system to the environment challenges, makes bioremediation an excellent method to clean phenolic compounds in an economical and friendly manner. Several microorganisms catalyze mineralization and/or transformation of hydroquinone either by aerobic or anaerobic processes, although the last one being less frequent. The knowledge of substrate specificity and toxicity patterns is needed in order to select strains with the required properties for efficient bioremediation. In fact, the inherent chemical properties of hydroquinone made it a poor substrate for classical phenol oxygenases, because the orientation of the two hydroxyl groups in *para* prevents further aromatic ring oxidations. Challenging the basic laws of chemistry, several microorganisms have developed extremely efficient enzymes which are able even to catalyze direct hydroquinone ring fission. Enzymatic systems designed for hydroquinone degradation are excellent examples of an elegant molecular design triggered by the natural selection. These enzymatic activities are promising tools for bioremediation of hydroquinone and its derivatives, and guided by the Synthetic Biology principles could be modeled, combined, and applied for targeted bioremediation of a wide family of phenolic compounds.

This review summarizes information on the biodegradation and biotransformation pathways of hydroquinone by different microorganisms as well as the recent development on its toxicity to mammalian and human cells. The characterization and mechanisms of action of the major hydroquinone detoxifying enzymes are also discussed. There are many factors, both biotic and abiotic, that influence the degradation of hydroquinone. Understanding and manipulating the flux of hydroquinone in the environment therefore require more holistic approaches, such as systems biology. It is known that each component involved in the biotransformation of hydroquinone and their interactions should be studied to provide the real scenario, which indeed constitutes a bottleneck to the scale up in order to achieve the desired outputs of bioremediation.

## Figures and Tables

**Table 1:** Hydroquinone properties.

Parameters		References
Other names	Dihydroxybenzene, 1,4-benzenediol, 1,4-dihydroxybenzene, *ρ*-benzenediol	—

Molecular weight	110.11 g/mol	—

Λ_max⁡_	289 nm	[25]

Melting point	169°C	[26]
	173-174°C	[27]

Boiling point	286°C	[26]
	287°C at 101.3 kPa	[28]

Density	1.300 kg/m^3^	[28]
	1.341 kg/m^3^	[26]
	1.332 kg/m^3^ at 15°C	[27]

Vapour pressure	2.34 × 10^−3^ Pa at 25°C	[26]
	2.40 × 10^−3^ Pa at 25°C	[27]

Partition coefficient (log⁡*P*_ow_)	0.50–0.61	[26]
	0.59	[28]

Water solubility	73 g/L at 25°C	[26]
	59 g/L at 25°C	[28]

pH	4.0–7.0	[26]

p*K*_*a*_	p*K*_1_ = 9.9	[26]
	p*K*_1_ = 9.9, p*K*_2_ = 11.6	[28]

Dipole moment	0.0 D	[28]

Polarity/polarizability parameter	0.21 ± 0.02 cm^3^	[28]

Standard electrode potential of half reaction for benzoquinone and hydroquinone	0.714 V	[29]
